# Evodiamine Induces G2/M Arrest and Apoptosis via Mitochondrial and Endoplasmic Reticulum Pathways in H446 and H1688 Human Small-Cell Lung Cancer Cells

**DOI:** 10.1371/journal.pone.0115204

**Published:** 2014-12-15

**Authors:** Chunshu Fang, Jingqing Zhang, Di Qi, Xiaoqing Fan, Jianchun Luo, Ling Liu, Qunyou Tan

**Affiliations:** 1 Department of Thoracic Surgery, Institute of Surgery Research, Daping Hospital, Third Military Medical University, Chongqing, People’s Republic of China; 2 Medicine Engineering Research Center, Chongqing Medical University, Chongqing, People’s Republic of China; Columbia University, United States of America

## Abstract

The goal of this study was to evaluate the ability of EVO to decrease cell viability and promote cell cycle arrest and apoptosis in small cell lung cancer (SCLC) cells. Lung cancer has the highest incidence and mortality rates among all cancers. Chemotherapy is the primary treatment for SCLC; however, the drugs that are currently used for SCLC are less effective than those used for non-small cell lung cancer (NSCLC). Therefore, it is necessary to develop new drugs to treat SCLC. In this study, the effects of evodiamine (EVO) on cell growth, cell cycle arrest and apoptosis were investigated in the human SCLC cell lines NCI-H446 and NCI-H1688. The results represent the first report that EVO can significantly inhibit the viability of both H446 and H1688 cells in dose- and time-dependent manners. EVO induced cell cycle arrest at G2/M phase, induced apoptosis by up-regulating the expression of caspase-12 and cytochrome C protein, and induced the expression of Bax mRNA and by down-regulating of the expression of Bcl-2 mRNA in both H446 and H1688 cells. However, there was no effect on the protein expression of caspase-8. Taken together, the inhibitory effects of EVO on the growth of H446 and H1688 cells might be attributable to G2/M arrest and subsequent apoptosis, through mitochondria-dependent and endoplasmic reticulum stress-induced pathways (intrinsic caspase-dependent pathways) but not through the death receptor-induced pathway (extrinsic caspase-dependent pathway). Our findings suggest that EVO is a promising novel and potent antitumor drug candidate for SCLC. Furthermore, the cell cycle, the mitochondria and the ER stress pathways are rational targets for the future development of an EVO delivery system to treat SCLC.

## Introduction

Lung cancer is the most common form of cancer, accounting for 12.5% of all annual newly diagnosed cancer cases worldwide. In addition to a high prevalence, lung cancer has the highest mortality rate among all cancer types [1]. Lung cancer can be classified into small-cell lung cancer (SCLC) and non-small-cell lung cancer (NSCLC) based on histopathological features of the disease. Approximately 10% to 15% of all lung cancers are SCLC [2]. Clinically, SCLC is distinguished from NSCLC by rapid tumor growth and widespread metastasis. According to the guidelines of the American Cancer Society [2], chemotherapy is the main treatment for SCLC, and cisplatin, etoposide, carboplatin and irinotecan are the most frequently used drugs. However, these drugs have only limited efficacy and cause severe side effects [3]. In fact, the five-year survival rate for SCLC is rather low (3∼8%) compared to the five-year survival rate for all forms of lung cancer (<15%) [4]. Novel and effective antitumor drugs with fewer and less severe side effects are urgently needed to improve the clinical outcomes.

Evodiamine (EVO), a major quinazolinecarboline alkaloid in *Evodia rutaecarpa*, has cytotoxic effects on different types of human cancer cells, such as glioblastoma cells [5], gastric cancer cells [6], breast cancer cells [7], bladder cancer cells [8] and lung cancer cells. Specifically, EVO exhibited cytotoxic effects on different NSCLC cell lines, including A549 lung adenocarcinoma cells [9], H1299 cells [10], CL1 cells [11] and H460 large-cell lung carcinoma cells [12], [13]. In contrast to the cytotoxic effects of EVO on various cancer cells [14], EVO has little effect on normal human peripheral blood cells or on the body weight of tumor-bearing mice at its effective dose [15].

On the other hand, some drugs that have cytotoxic effects on NSCLC also have obvious effects on SCLC, such as wentilactone A, which is cytotoxic to both H460 and H446 [16], and glucosamine, which is cytotoxic to A549 and H446 cells [17]. Therefore, although there has been no report of the effect of EVO on SCLC to date, EVO may be a potential new drug candidate for the treatment of SCLC.

In this study, the effects of EVO on cell viability, the cell cycle and apoptosis in the human SCLC cell lines NCI-H446 and NCI-H1688 were investigated, and the underlying mechanisms was further explored. Our results indicated that the inhibitory effects of EVO on the growth of H446 and H1688 cells were attributable to G2/M arrest and subsequent apoptosis through the mitochondria-dependent and endoplasmic reticulum (ER) stress-induced caspase activation pathways (intrinsic caspase-dependent pathways) but not through the death receptor-induced caspase activation pathway (extrinsic caspase-dependent pathway). So far, no drug has been reported to induce apoptosis in SCLC cells via ER stress pathway. Our results represent the first report that EVO induces apoptosis in the H446 and H1688 SCLC cell lines via the ER stress pathway. In addition, there has been no previous report of a drug that simultaneously induces cell cycle arrest and apoptosis in SCLC cells via mitochondria-mediated and ER stress pathways. We report for the first time that EVO induced G2/M arrest and apoptosis via both the mitochondria-mediated and ER stress pathways in the H446 and H1688 SCLC cell lines.

## Materials and Methods

### 2.1 Compound

Evodiamine (EVO) was purchased from Yuancheng Technology Development Co., Ltd. (Wuhan, China), purity 99.13%. EVO was dissolved in dimethylsulfoxide (DMSO) to prepare a 40 mM stock solution, which was diluted with Roswell Park Memorial Institute (RPMI) 1640 medium (Grand Island Biological Company, Grand Island, NY, USA) containing 10% fetal bovine serum (FBS) before every experiment. The final DMSO concentration was not more than 0.025% in this study.

### 2.2 Cell Culture

The human NCI-H446 and NCI-H1688 SCLC cell lines were purchased from the Chinese Academy of Medical Sciences (Beijing, China) and the Chinese Academy of Sciences (Shanghai, China), respectively. H446 and H1688 cells were cultured in RPMI 1640 medium supplemented with 10% FBS, 100 IU/mL of penicillin and 100 µg/mL of streptomycin (Gibco Co., Grand Island, NY, USA). The cells were incubated at 37°C in a humidified atmosphere containing 5% CO_2_.

### 2.3 Cell Viability Assays

The H446 or H1688 cells were seeded at a density of 2×10^3^ cells/well in 96-well microplates (Corning Incorporated, NY, USA). The cells were grown for 12 h, and the medium was replaced with RPMI 1640 media containing different concentrations of EVO (1.25, 2.5, 5, 10 and 20 µM). After the end of the specified incubation period (24 h, 48 h and 72 h), the medium was exchanged with fresh medium containing 20 µL of 5 mg/mL methyl thiazolyl tetrazolium (MTT) solution (Sigma). After incubation for 4 h, the MTT solution was removed and replaced with 150 µL of DMSO, and the microplates were shaken for 5 min. The absorbance was measured at 490 nm with a Multiskan GO Microplate Spectrophotometer (Thermo Fisher Scientific, Inc., Waltham, MA, USA). The cell viability was calculated as follows: Cell viability (%) = OD_test group_/OD_control group_×100%, where OD_test group_ was the optical density (OD) of the EVO or DMSO treatment group and OD_control group_ was the optical density of the negative control group. Untreated H446 or H1688 cells were used as a negative control group. The IC_50_ value refers to the concentration of drug required to kill 50% of the cells [18]. The cell viabilities of different EVO concentrations were analyzed by OriginPro 7 software (OriginLab Corporation, Northampton, MA, USA), and then the IC_50_ values were obtained. The morphologies of H446 cells incubated with EVO for 24 h were visualized under an inverted fluorescence microscope (ECLIPSE Ti-S, Nikon Instruments Inc., Tokyo, Japan).

### 2.4 Cell Cycle and Apoptosis Analyses

The H446 or H1688 cells were cultured in 25 cm^2^ flasks and treated with 10 µM EVO for 24 h. The cells were harvested by trypsinzation and centrifugation, and then fixed with 70% ethanol at 4°C for 12 h. After rinsing twice with phosphate-buffered solution (PBS), the cells were resuspended in a DNA staining solution containing 40 µg/mL propidium iodide (PI) and 0.1 mg/mL RNase at 25°C in the dark for 30 min. The cells were analyzed with a FACSVantage flow cytometer (Becton-Dickinson, San Jose, CA, USA) equipped with the CellQuest software [18]. Then, the cell cycle distribution was determined and analyzed.

Apoptotic cell quantification was determined using an Annexin V-FITC/PI apoptosis detection kit (Beyotime Institute of Biotechnology, Shanghai, China). The induction of apoptosis was detected in H446 or H1688 cells after 24 h of treatment with 10 µM EVO according to the Annexin V-FITC/PI staining method. The H446 cells were observed under a Nikon Eclipse Ti inverted fluorescence microscope.

### 2.5 Reactive Oxygen Species (ROS), Intracellular Free Calcium (Ca^2+^), and Mitochondrial Membrane Potential (ψ_m_)

ROS, Ca^2+^ and ψ_m_ levels were determined with a FACSVantage flow cytometer using the following three fluorochromes: 2′,7′-dichlorofluorescin diacetate (DCF-DA) (Beyotime Institute of Biotechnology, Haimen, Jiangshu, China), Fluo-3/AM (Beyotime Institute of Biotechnology, Shanghai, China), and JC-1 (Beyotime Institute of Biotechnology, Jiangshu, China), respectively [19]. Briefly, H446 or H1688 cells seeded at a density of 1×10^6^ cells/well in 6-well plates (Corning Incorporation, NY, USA) were treated with 10 µM EVO for 24 h. The cells were collected, centrifuged and resuspended in a staining solution containing 10 µM DCF-DA (5 µM Fluo-3/AM or 5 µg/mL JC-1) at 37°C for 30 min (45 min or 20 min) and then analyzed using a FACSVantage flow cytometer.

### 2.6 Caspase-3, -8, and -9 Activity Assay

The caspase-3,-8 and -9 activity levels were measured using activity assay kits (Beyotime Institute of Biotechnology, Haimen, Jiangsu, China). Briefly, H446 or H1688 cells were harvested after being treated with 10 µM EVO for 24 h (48 h or 72 h). Then, the cells were washed with cold PBS, re-suspended in lysis buffer (100 µL per 2×10^6^ cells), left on ice for 15 min and then centrifuged at 18,000×*g* at 4°C for 10 min. The assays were performed in 96-well microtitre plates by incubating a mixture composed of 10 µL of the cell lysate, 80 µL of reaction buffer and 10 µL of caspase-3 (-8 or -9) substrate (Ac-DEVD-pNA) at 37°C for 4 h. The caspase-3 (-8 or -9) activity in the samples was quantified using a Multiskan GO Microplate Spectrophotometer (Thermo Fisher Scientific Inc., Waltham, MA, USA) at an absorbance of 405 nm.

### 2.7 Western Blot Analysis

Cytochrome C (Cyt C), caspase-12, -8, -9 and -3, factor associated suicide (Fas) and tumor necrosis factor-related apoptosis inducing ligand (Trail) were measured at the protein level by Western blotting. H446 cells treated with 10 µM EVO for 48 h were collected and incubated in radio immunoprecipitation assay (RIPA) lysis buffer (Beyotime Institute of Biotechnology, Haimen, Jiangshu, China) for 60 min on ice. The cell lysates were centrifuged at 13000 g for 15 min, and the protein concentrations in the lysates were determined using the Bio-Rad protein assay Dye (Bradford) Reagent (Bio-Rad Laboratories, Hercules, CA, USA). Equal amounts of proteins were resolved by sulfate-polyacrylamide gel electrophoresis (SDS-PAGE) and blotted onto Immobilon-P transfer membranes (Millipore Corporation, Bedford, MA, USA).

The membranes were blocked with 5% nonfat milk in TBST buffer (20 mM Tris-HCl, 150 mM NaCl and 0.05% Tween 20). Cyt C, caspase-12, -8, -9 and -3, Fas and Trail were detected using primary antibodies (rabbit anti-Cyt C, caspase-12, -8, -9 and -3, Fas and Trail) and secondary antibodies (goat anti-rabbit IgG(H+L), horseradish peroxidase-conjugated). All the antibodies were purchased from Beijing Biosynthesis Biotechnology Co., LTD., Beijing, China and they were diluted 1∶200 with 5% skim milk TBST (Sigma) before use. The final concentration of the antibodies was 20 µg/mL. Similarly, Cyt C and caspase-12 and -8 were measured in H1688 cells treated with EVO for 48 h by Western blotting.

### 2.8 Reverse Transcription Polymerase Chain Reaction (RT-PCR)

Total cellular RNA from freshly isolated H446 cells was isolated using TRIzol reagent (Invitrogen, Carlsbad, CA, USA). The cDNA was synthesized using reverse transcriptase (Genecopoeia Inc., Rockville, MD, USA). The specific gene product was amplified by PCR with Taq DNA polymerase (Fermentas, Waltham, MA, USA). The primer sets for PCR were as follows:

Bax sense strand: 5′-TTTGCTTCAGGGTTTCATCCA-3′;

Bax antisense strand: 5′-CCAGCCTTGAGCACCAGTTT-3′.

Bcl-2 sense strand: 5′-ACTTCGCCGAGATGTCCAGC-3′;

Bcl-2 antisense strand: 5′-GCACCTACCCAGCCTCCGTTAT-3′;

### 2.9 Statistical Analysis

Each experiment in this study was repeated 3 times. All data are shown as the mean ± standard deviation (SD) unless stated otherwise. The analyses were performed using the Statistical Package for the Social Sciences (version 13.0, SPSS Inc., Chicago, IL, USA). Student’s t test was used to determine the statistical significance of the differences among the experimental groups. Statistical significance was established at *P*<0.05.

## Results

### 3.1 Inhibitory Effects of Evodiamine on the Cell Growth

**Figure 1:**
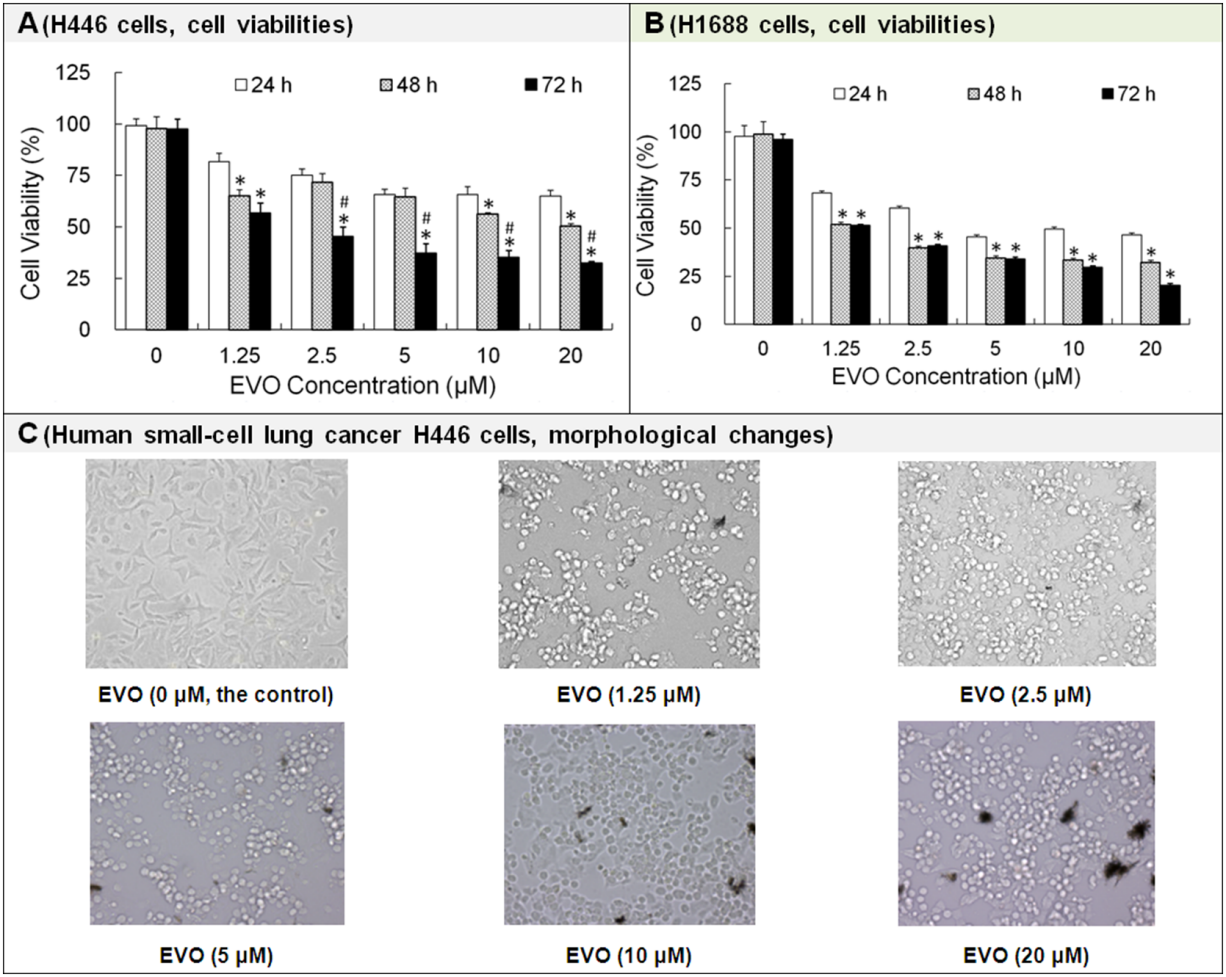
Effects of evodiamine (EVO) on the viability of H446 and H1688 cells. The cell viability was measured by MTT assay. The cells were photographed using microscope. Each experiment was repeated 3 times. Data presented as mean ± standard deviation (n = 3). *P<0.05 showed significant difference between two groups. Untreated H446 or H1688 cells were used as a negative control group. The 0 µM EVO group contained 0.025% DMSO. The 0.025% DMSO was used to prepare 20 µM EVO (the maximum concentration of EVO solution in the study). *P<0.05 as compared to the corresponding EVO treated group at 24 h. ^#^ *P*<0.05 as compared to corresponding EVO treated group at 48 h.

The inhibitory effects of EVO on human SCLC H446 and H1688 cell growths were evaluated using MTT cytotoxicity assays. As shown in **Fig. 1A**, EVO inhibited human SCLC H446 growth in a dose- and time-dependent manner. The viability rate of H446 cells treated with 10 µM EVO for 24 h (∼66%) decreased by ∼16% compared to that for cells treated with 1.25 µM EVO (∼82%). The viability rate of H446 cells treated with 10 µM EVO for 72 h (∼35%) decreased by ∼22% compared to that for cells treated with 1.25 µM EVO (∼57%). The IC_50_ values for EVO-treated H446 cells for 24 h, 48 h and 72 h were >20 µM, 18.07 µM and 1.80 µM, respectively.

EVO inhibited human SCLC H1688 cell growth in a slightly different manner (**Fig. 1B**): (1) EVO inhibited human SCLC H1688 cell growth in a dose-dependent manner within 72 h. The viability rates of H1688 cells treated with 10 µM EVO (∼49% at 24 h, ∼33% at 48 h and ∼30% at 72 h, respectively) decreased by ∼19%, 19% and 21% compared to that treated with 1.25 µM EVO (∼68% for 24 h, ∼52% for 48 h and ∼51% for 72 h, respectively). (2) EVO inhibited H1688 growth in a time-dependent manner at concentrations ranging from 1.25 µM to 10 µM within 48 h and at a concentration of 20 µM within 72 h. (3) The inhibition rates after treatment for 48 h were almost the same as those after 72 h of treatment with EVO at concentrations ranging from 1.25 µM to 10 µM. (4) The IC_50_ values of EVO in H1688 cells decreased from 8.14 µM (at 24 h) to 2.08 µM (at 48 h) or 1.37 µM (at 72 h).

After treatment with 10 µM EVO, the viability rates of H446 or H1688 cells were ∼66% or ∼49% (24 h), ∼56% or ∼33% (48 h), and 35% or ∼30% (72 h), respectively. Because of the obvious inhibitory effect of 10 µM EVO on H446 or H1688 cells, the intermediate dose of EVO (i.e., 10 µM) was selected to be used in all the following tests unless otherwise noted.

**Fig. 1C** indicated that the morphologies of H446 cells treated with different concentrations of EVO for 24 h were substantially altered. When the EVO concentration increased, more H446 cells became round and detached from the culture plate as their pseudopodia gradually retracted. EVO inhibited H446 cell growth in a dose-dependent manner, except that H446 cells treated with EVO at 5 µM, 10 µM or 20 µM for 24 h had similar cytotoxicity effects.

Taken together, the natural herbal constituent EVO significantly inhibited the viabilities of H446 and H1688 SCLC cells in dose- and time-dependent manners.

### 3.2 Effects of Evodiamine on Cell Cycle and Apoptosis

**Figure 2:**
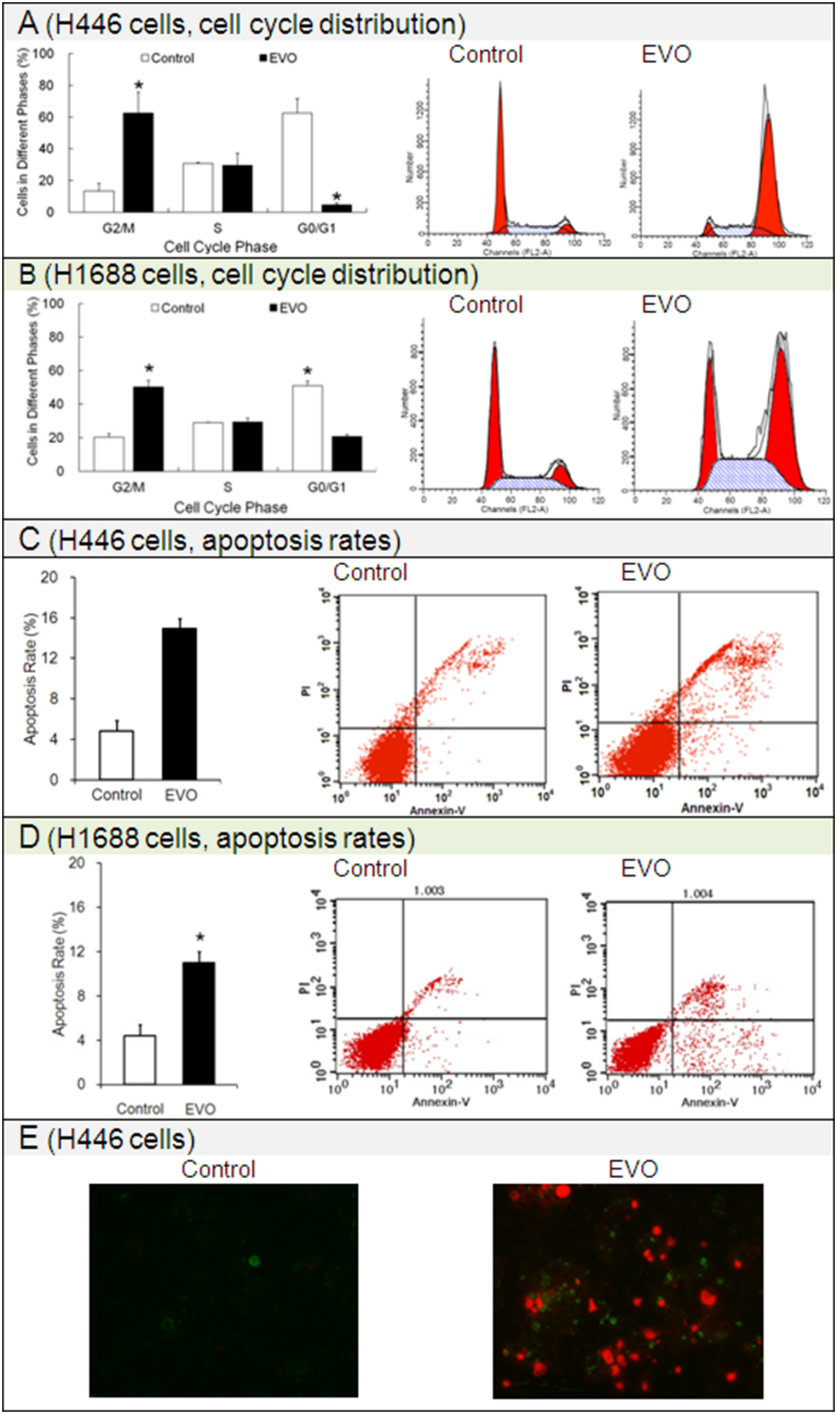
Effects of evodiamine (EVO) on the cell cycle distribution and apoptosis rate of the H446 and H1688 SCLC cells. Cell cycle was detected by PI assay. Apoptosis was detected using an Annexin V/PI double staining assay. The H446 cells stained with Annexin V/PI were observed under an inverted fluorescence microscope. Each experiment was repeated 3 times. Data presented as mean ± standard deviation (n = 3). *P<0.05 as compared to corresponding control group. Untreated H446 or H1688 cells were used as a negative control group.

To examine disruption of the cell cycle was responsible for the EVO-mediated cell growth inhibition, we studied the cell-cycle distribution. As shown in **Fig. 2A and 2B**, EVO selectively arrested the cell cycle at G2/M phase (i.e., the pre-mitotic/mitotic phase). After treatment with 10 µM EVO for 24 h, the number of EVO-treated H446 or H1688 cells in G2/M (∼63% or ∼50%) was approximately 5-fold or 2.5-fold that of the untreated cells (blank control, ∼13% or 20%). Meanwhile, the numbers (∼31% or ∼29%) of EVO-treated H446 or H1688 cells in S phase (the synthesis phase during which the chromosomes are replicated) were almost the same as those of the untreated cells (∼30% or ∼29%).

Apoptosis is also referred to as cellular suicide or programmed cell death. To determine whether EVO induced apoptosis in SCLC H446 and H1688 cells, the apoptosis rates were detected by Annexin V-FITC/PI double staining. After treatment with EVO for 24 h, as indicated in **Fig. 2C and 2D** **,** the apoptosis rate of EVO-treated H446 (∼15%) or H1688 (∼11%) cells was much higher than that of the untreated cells (blank control, ∼5% or ∼4%); as shown in **Fig. 2E**, typical features of apoptosis, such as chromatin condensation and marginalization, nuclear segmentation and apoptotic body formation, were observed in EVO-treated H446 cells. In short, EVO significantly induced apoptosis in both H446 and H1688 cells. It should be noted that in our preliminary experiments, we assessed the apoptotic effects of lower doses of EVO (such as 1.25 µM and 2.5 µM). The apoptosis rates of SCLC H446 cells treated with 1.25 µM EVO or 2.5 µM EVO were almost the same as those of the corresponding blank controls (data not shown).

### 3.3 Effects of Evodiamine on ROS, Ca^2+^ and ψ_m_ Levels

**Figure 3:**
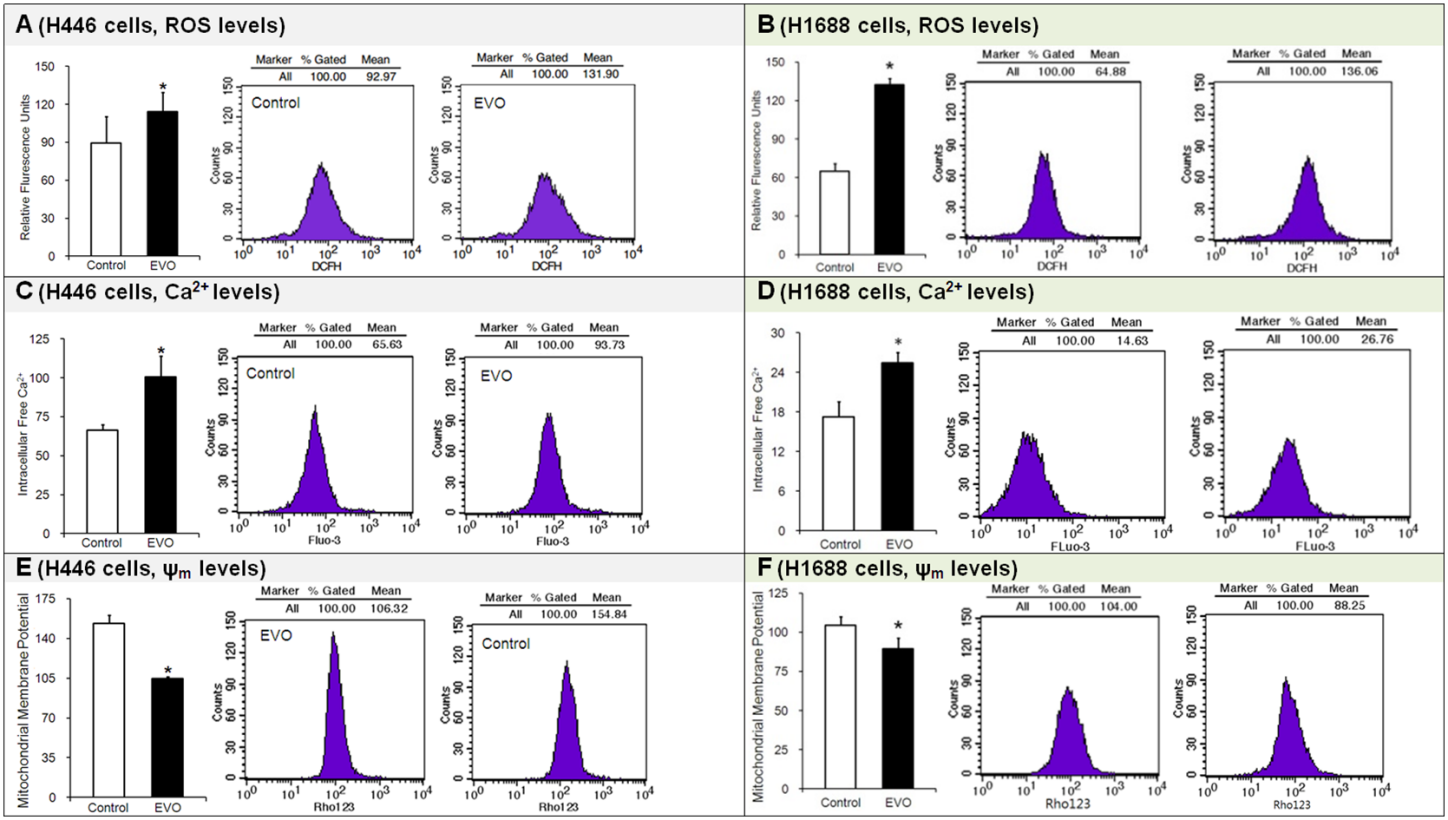
Effects of evodiamine (EVO) on the levels of ROS, Ca^2+^ and ψ_m_ in H446 and H1688 SCLC cells. ROS, Ca^2+^ and ψ_m_ were separately detected by DCF-DA, Fluo-3/AM and JC-1 assays. Each experiment was repeated 3 times. Data presented as mean ± standard deviation (n = 3). Untreated H446 or H1688 cells were used as a negative control group. *P<0.05 as compared to corresponding control group.

The ROS, Ca^2+^ and ψ_m_ levels were critical factors indicating the possible activation of apoptosis pathways. Most ROS are free radicals that cause DNA, protein and biomembrane damage [18]. Intracellular calcium is a major intracellular signaling factor. Ψ_m_ is a key indicator of cell health because it is related to the ATP generation capacity of cells. In this study, the ROS, Ca^2+^ and ψ_m_ were measured with fluorescent probes using a FACSVantage flow cytometer [18]. After treatment with EVO for 24 h, compared with the control groups, (1) the ROS levels in EVO-treated SCLC H446 cells increased by more than one-quarter (∼28%), and in H1688 cells, the ROS levels more than doubled (∼104%) (**Fig. 3A and 3B**); (2) the intracellular Ca^2+^ levels in both the H446 and H1688 cells increased by half (∼51% or ∼48%) (**Fig. 3C and 3D**); (3) the ψ_m_ levels decreased by almost one-third (∼32%) and by one-seventh (∼14%) in the H446 and H1688 cells, respectively (**Fig. 3E and 3F**). The results suggest that EVO increased the ROS levels in SCLC cells. Excess ROS not only damaged the DNA, but it also damaged the mitochondrial membrane and increased mitochondrial membrane permeability. More calcium was released from the mitochondria and entered the cytoplasma. The intracellular calcium concentration increased, and the mitochondrial membrane potential was partially depolarized. EVO induced apoptosis in both SCLC H446 and H1688 cells through the ROS-mediated mitochondrial pathway.

### 3.4 Effects of Evodiamine on Caspase-8, -9 and -3 Activity Assay

**Figure 4:**
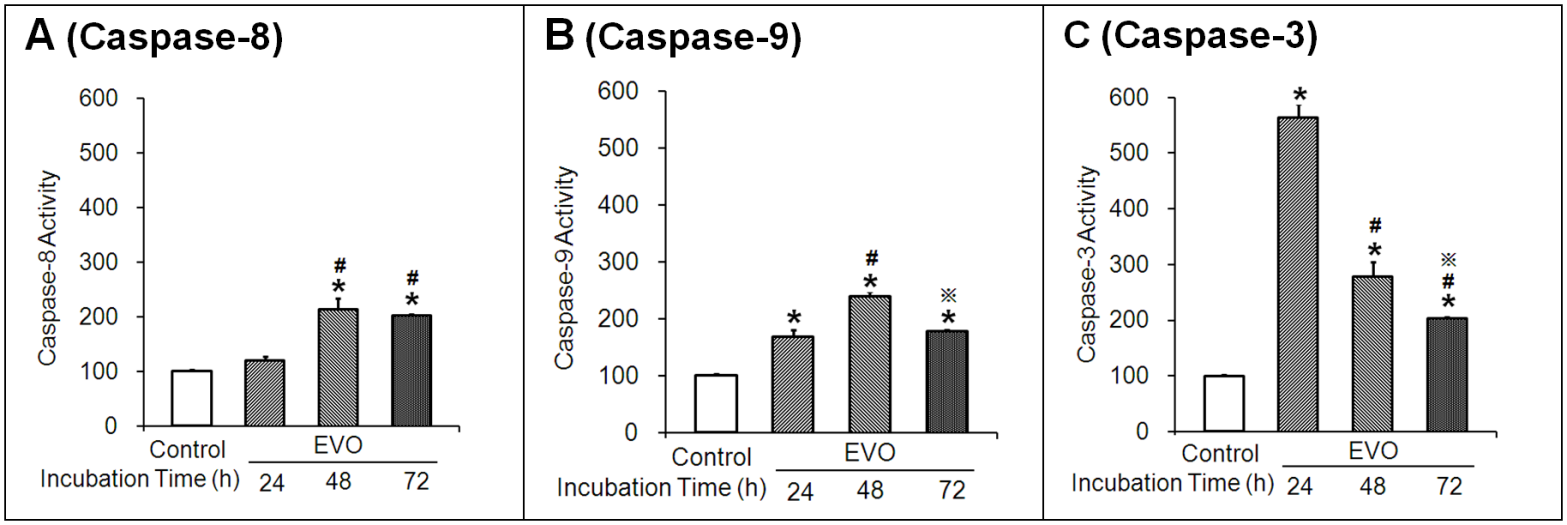
Effects of evodiamine (EVO) on the activities of caspase-8 (A), -9 (B) and -3 (C) in H446 cells. Cell lysates were analyzed by a colorimetric assay of Ac-DEVD-pNA. Each experiment was repeated 3 times. Data presented as mean ± standard deviation (n = 3). Caspase activities were given as arbitrary units (AU) per milligram of protein. Untreated H446 cells were used as a negative control group. *P<0.05 as compared to the corresponding control group. ^#^ *P*<0.05 as compared to corresponding EVO treated group at 24 h. ^
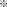
^ *P*<0.05 as compared to corresponding EVO treated group at 48 h.

Caspases are proteolytic enzymes that are critical mediators of apoptosis. The activities of caspase-8, -9 and -3 were determined by spectrophotometry. Compared to the corresponding control groups, significant increases in the activities of caspase-8, -9 and -3 were found in H446 cells treated with 10 µM EVO for 24 h, 48 h and 72 h, with the exception of the increase in caspase-8 activity in EVO-treated cells after 24 h, which was not statistically significant. The highest levels of caspase-8 (∼213%**,** **Fig. 4A**) and caspase-9 activity (∼240%, **Fig. 4B**) were observed after treatment with EVO for 48 h, while the highest level of caspase-3 activity (∼564%) was observed at 24 h (**Fig. 4C**). However, the highest caspase-8 and -9 activities were still lower than the caspase-3 activity (∼278%) in EVO-treated cells at 48 h. These results suggest that EVO induced apoptosis through caspase-dependent pathways.

### 3.5 Effects of Evodiamine on the Protein Expression of Cyt C, Caspase-12, -8, -9 and -3, Fas and Trail

Cyt C is a mitochondrial protein involved in the initiation of mitochondria-mediated apoptosis. Caspases are cysteine-aspartic acid proteases that are essential for apoptosis. Caspase-12 is localized to the ER and involved in ER-mediated apoptosis. Caspase-8 mediates signal transduction downstream of death receptors (DR) located on the plasma membrane and is thus involved in DR-mediated apoptosis. The interaction of DR with its natural ligands (such as FasL and Trail) induces the activation of caspase-8. Caspase-9 is an aspartic acid-specific protease. Caspase-3 is an executioner caspase responsible for chromatin condensation and DNA fragmentation in apoptosis. Cyt C and caspase-12 and -8 trigger the activation of caspase 9 (initiator caspase) and caspase-3 (effector caspase) before they finally induced apoptosis.

**Figure 5:**
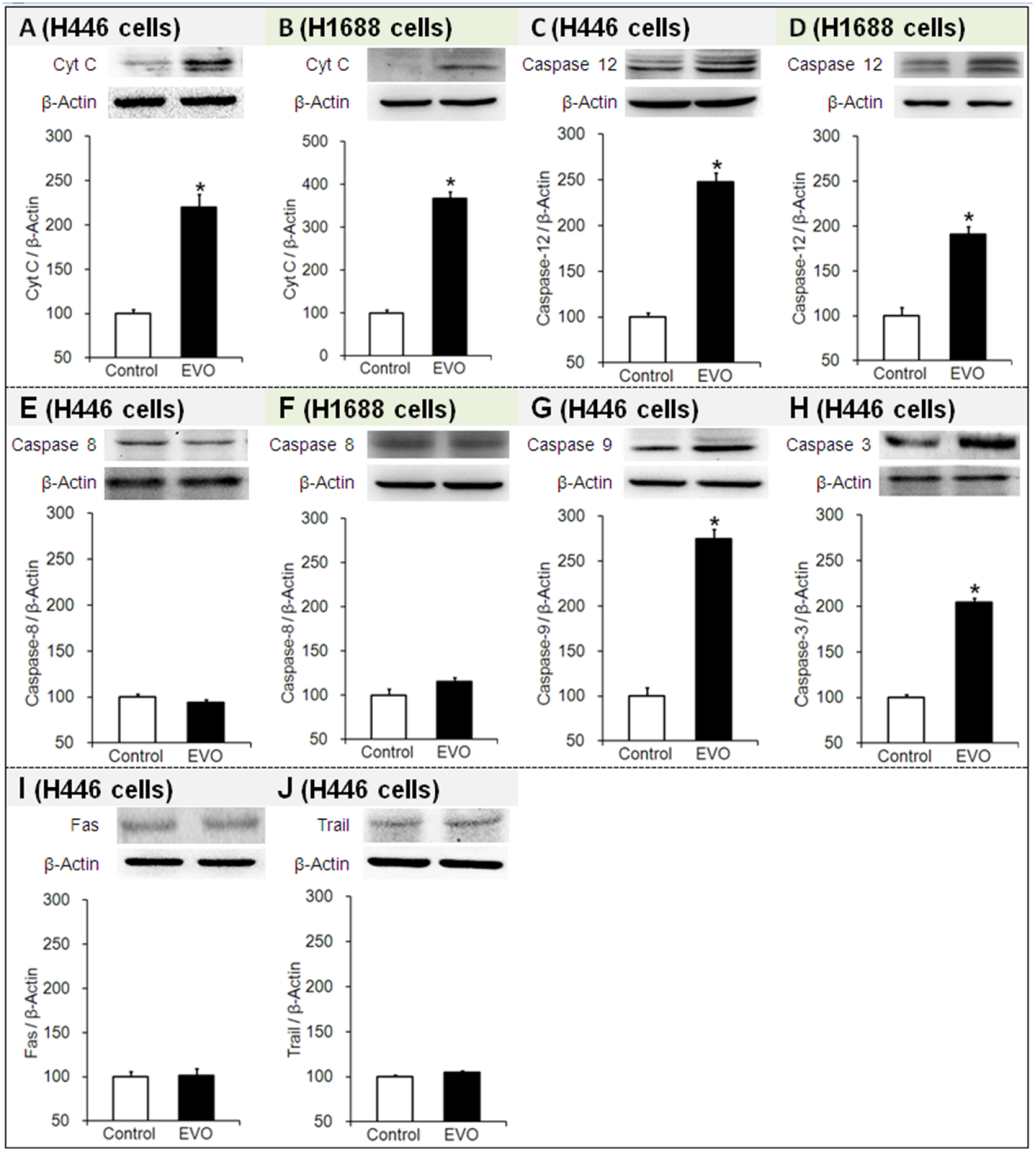
Effects of evodiamine (EVO) on the protein expression of Cyt C, caspase-12, -8, -9 and -3, Fas and Trail in the H446 and H1688 SCLC cells. Cell lysates were analyzed by Western blot. Each experiment was repeated 3 times. Data presented as mean ± standard deviation (n = 3). Untreated H446 or H1688 cells were used as a negative control group. *P<0.05 as compared to corresponding control group. Fas: factor associated suicide; Trail: tumor necrosis factor-related apoptosis inducing ligand; Cyt C: cytochrome C.

For EVO-treated H446 or H1688 cells, compared to their corresponding control groups (the level was set as 100% in each of the controls), (1) the protein expression levels of Cyt C were significantly increased by ∼220% and ∼367% (see **Fig. 5A and 5B**), respectively, (2) the protein expression levels of caspase-12 were significantly increased by ∼248% and ∼190% (see **Fig. 5C and 5D**), respectively, and (3) the protein expression levels of caspase-8 were almost unchanged (∼94% and ∼112%, respectively) (see **Fig. 5E and 5F**). Further investigation of H446 cells treated with EVO revealed that the marked elevation of the level of Cyt C resulted in increased caspase-9 and -3 expression by ∼275% and 204%, respectively (see **Fig. 5G and 5H**). Furthermore, FasL and Trail, which are caspase-8 activators, were not unchanged (∼102% and ∼105%, respectively) (see **Fig. 5I and 5J**). The western blot results suggested that EVO increased the Cyt C and caspase-12 levels in both H446 and H1688 SCLC cells, thus EVO induced apoptosis through both the mitochondria- and ER-mediated pathways. Furthermore, the elevated protein expression of caspase-9 and -3 indicated that EVO induced apoptosis through a caspase-3-dependent pathway. Contrarily, EVO had no effect on the protein expression of caspase-8 in either H446 or H1688 SCLC cells, which suggests that it did not induce apoptosis through a DR-mediated pathway.

### 3.6 Effects of Evodiamine on the mRNA Expression of Bax and Bcl-2

**Figure 6:**
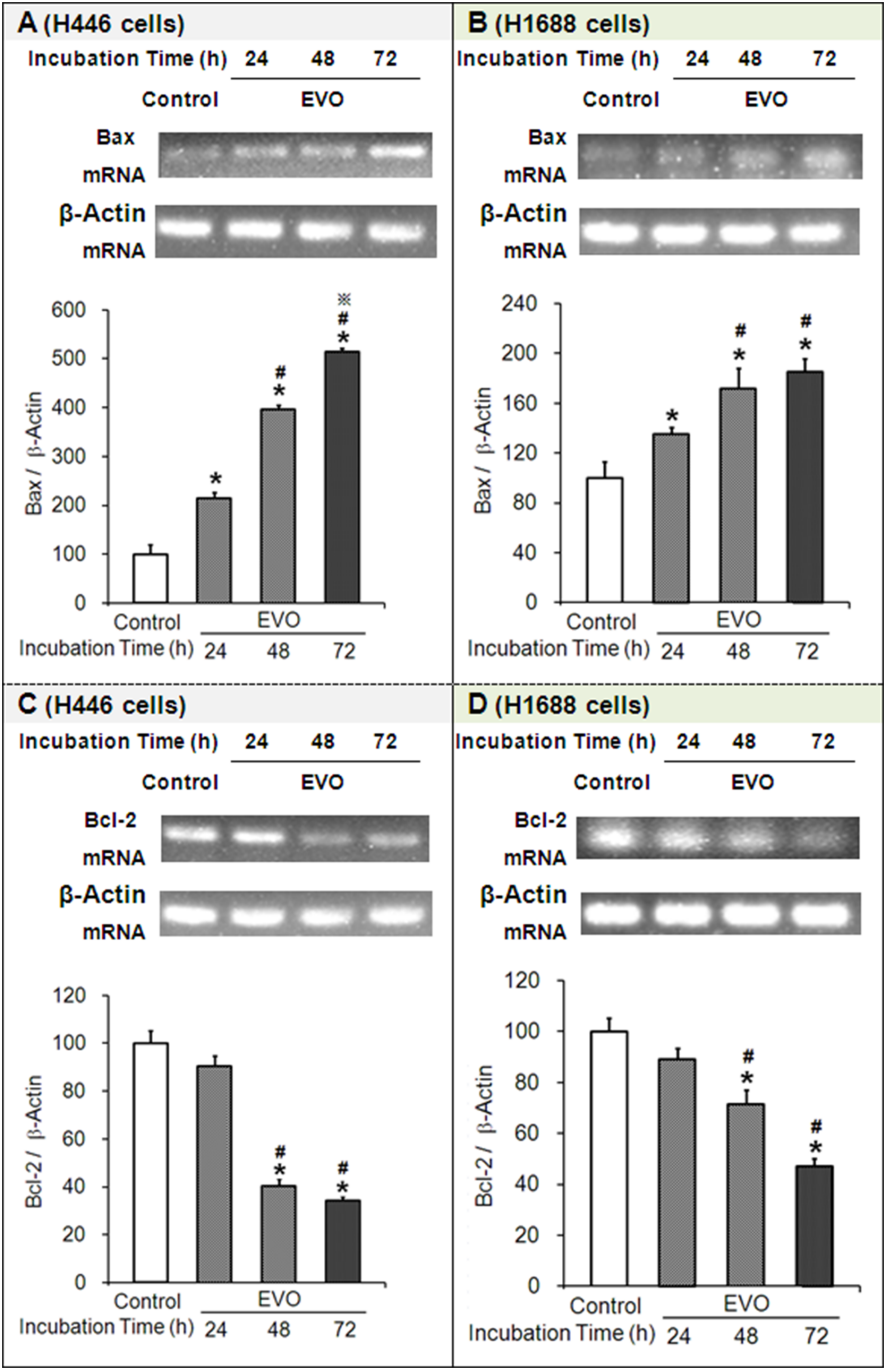
Effects of evodiamine (EVO) on the mRNA expression of Bax and Bcl-2 in H446 and H1688 cells. Cell lysates were analyzed by RT-PCR. Each experiment was repeated 3 times. Data presented as mean ± standard deviation (n = 3). Untreated H446 or H1688 cells were used as a negative control group. *P<0.05 as compared to the control group. ^#^ *P*<0.05 as compared to corresponding EVO treated group at 24 h. ^
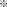
^ *P*<0.05 as compared to corresponding EVO treated group at 48 h.

BAX is also known as Bcl-2-like protein 4 or Bcl-2-associated X; Bcl-2 stands for B-cell lymphoma 2. Bax promotes apoptosis by antagonizing Bcl-2, which is specifically considered an important anti-apoptotic protein. Simultaneously, BAX and Bcl-2 are separately encoded by the BAX and Bcl-2 genes. Here, the expression levels of the Bax and Bcl-2 genes were determined by RT-PCR. Compared to their corresponding controls, (1) the mRNA expression levels of Bax in H446 or H1688 cells treated with EVO were significantly increased by ∼215% or ∼135% (at 24 h), ∼397% or ∼172% (at 48 h) and ∼514% or ∼185% (at 72 h), respectively (**Fig. 6A and 6B**). (2) On the contrary, the mRNA expression levels of Bcl-2 in EVO-treated H446 or H1688 cells were significantly decreased by ∼10% or ∼11% (at 24 h), ∼60% or ∼28% (at 48 h), and ∼66% or ∼53% (at 72 h), respectively (**Fig. 6C and 6D**). Taken together, EVO increased the ratio of Bax/Bcl-2 at the transcriptional level, which is expected to further increase the ratio of Bax/Bcl-2 at the protein level and eventually promote apoptosis.

## Discussion

In this study, we show for the first time that EVO significantly inhibits the viability of SCLC cells. The IC_50_ values of EVO in H446 cells decreased from >20 µM (24 h) to 18.07 µM (48 h) or 1.80 µM (72 h); and in H1688 cells, the IC_50_ decreased from 8.14 µM (24 h) to 2.08 µM (48 h) or 1.37 µM (72 h). EVO exerted inhibitory effects on H446 and H1688 cells in concentration- and time-dependent manners. It was previously documented that the IC_50_ values of EVO in human NSCLC cells were 12 µM (48 h, H460 cells) [13], 13.2 µM (72 h, (R)-EVO, H460 cells) [12], 2.6 µM (72 h, (S)-EVO, H460 cells) [12], and 100 µM (72 h, A549 cells) [9]. In short, EVO exhibited antitumor activity in SCLC in addition to NSCLC cells. Furthermore, in most cases, EVO inhibited the growth of H446 SCLC cells more efficiently than that of H460 and A549 NSCLC cells.

The investigation of the cell cycle distribution demonstrated that disruption of the cell cycle was responsible for EVO-mediated cell growth inhibition. The number of H446 and H1688 cells in S phase was almost unchanged after EVO treatment compared to the control. However, EVO triggered an arrest at G2/M phase, i.e., EVO-treated H446 and H1688 cells accumulated in the G2/M phase. An arrest of cells in G2/M phase in response to genotoxic stress (such as oxidative stress and DNA intercalating agents) might induce DNA damage in both a p53-dependent (via ROS) [20] and p53-independent manner [21].

**Figure 7:**
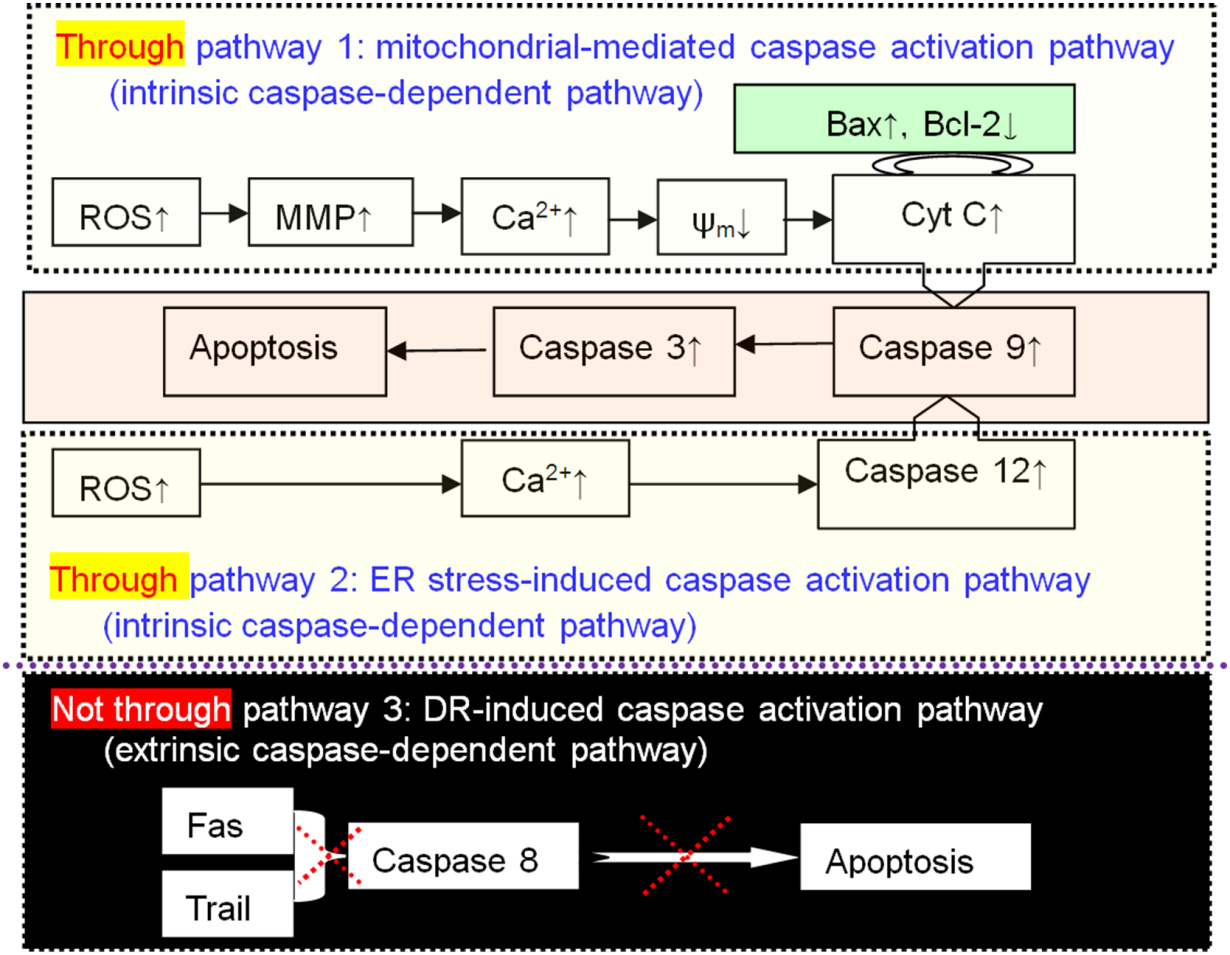
Evodiamine (EVO) induces apoptosis through two intrinsic caspase-dependent pathways, but not through an extrinsic caspase-dependent pathway.

It was previously documented that EVO induced apoptosis in different human cancer cells via different pathways. For example, in NSCLC H1299 cells apoptosis was induced through suppression of the nuclear factor (NF)-κB activation pathway [10]; and in pancreatic SW1990 cells, apoptosis was induced via regulation of the PI3 K/Akt pathway [22], in 253J and T24 bladder cancer cells, apoptosis was induced through mTOR/S6K1-mediated downregulation of Mcl-1 [8]. Apoptosis induction by EVO in H446 or H1688 SCLC cells was characterized by Annexin V-FITC/PI double staining, and morphological changes were evident in H446 cells. Additionally, the results of the present study suggest that G2/M phase cell cycle arrest halted H446 and H1688 cell growth and eventually led to cell death by apoptosis through two intrinsic caspase-dependent pathways, but not through the extrinsic caspase-dependent pathway (**Fig. 7**):

1. Apoptosis was induced through the mitochondria-mediated caspase activation pathway. The results showed that EVO triggered mitochondrial apoptosis accompanied by the accumulation of ROS. In this study, the change of mitochondrial membrane permeability induced by ROS generation led to the activation of intracellular calcium release, the loss of mitochondrial membrane potential, and the subsequent release of Cyt C. Being an apoptosis factor, Cyt C further triggered the activation of caspase 9 (initiator caspase) followed by the activation of caspase-3 (effector caspase), the induction of PARP cleavage and the final induction of apoptosis [23]. It was previously documented that piperine [24] (or benzamide riboside [25]) triggered mitochondria-mediated apoptosis in A549 (or H520) NSCLC cells via a p53-dependent signaling pathway, and 20(S)-protopanaxadiol induced mitochondria-mediated apoptosis in A549 cells by inhibiting the PI3 K/Akt signaling pathway [26].

It was previously reported that tanshinone IIA inhibited the growth of H146 SCLC cells by up-regulating the Bax/Bcl-2 ratio and decreasing the mitochondrial membrane potential [27]. Furthermore, curcumin induced apoptosis in H446 SCLC cells via the ROS-mediated mitochondrial pathway and by increasing Bax expression while decreasing the expression of Bcl-2 and Bcl-xL [28]. Erlotinib induced mitochondria-mediated apoptosis in H3255 NSCLC cells through mitochondrial oxidative phosphorylation-dependent activation of Bax and Bak [29]. In our study, the effects of EVO on the mRNA expression of Bax and Bcl-2 were evaluated by RT-PCR. Bax was upregulated and derepressed, while Bcl-2 was downregulated and repressed. Briefly, EVO treatment increased the ratio of Bax/Bcl-2 expression, which played an important role in mediating cell apoptosis and survival. It is widely recognized that the Bcl-2 family plays critical roles in apoptosis, largely by regulating the mitochondrial transmembrane potential ψ_m_ [30]. EVO significantly induced the accumulation of ROS and the dissipation of mitochondrial ψ_m_ in H446 or H1688 cells. The changes in the expression levels of Bax and Bcl-2 (pro-apoptotic and anti-apoptotic genes of the Bcl-2 family) during EVO treatment might be due to the increased phosphorylation and accumulation of p53, as previously validated by LV et al. [16].

1. Apoptosis was induced by the ER stress-induced caspase activation pathway. It was previously reported that palladium bis-acetylacetonate [31], propofol [32], and a curcumin analog [33] induced apoptosis in H460 NSCLC cells via the ER stress pathway. Anacardic acid [34] had a similar effect in A549 cells, as did furanodiene in 95-D cells [35]. There has been no previous report of a drug that induces apoptosis in SCLC cells via the ER stress pathway. For the first time, we have reported that EVO triggers ER stress-induced apoptosis in H446 SCLC cells. EVO induced the activation of the ER specific caspase 12, and the cleavage of procaspase 12 further led to the activation of caspase 9 and 3 in EVO treated H446 and H1688 cells. The ER is a principle intracellular calcium store. The efflux of Ca^2+^ from ER stores could be regulated by PLCγ, a critical enzyme that might be activated by ER stress (here, the increased ROS stimulated the signaling) [36]. The efflux of Ca^2+^ from ER stores could be regulated by PLCγ, a critical enzyme that might be activated by ER stress (here, the increased ROS stimulated the signaling) [36]. In 2013, Schönthal reviewed the pharmacological targeting of ER stress signaling in cancer [37]. Xu et al. proposed that the chemotherapeutic efficiency of cisplatin could be enhanced by targeting ER stress in some cancer cells, such as A549 NSCLC cells [38]. Cisplatin is one of the most commonly used drugs for the treatment of SCLC, but until now, there has been no experimental data to support the hypothesis that cisplatin triggered ER stress-induced apoptosis in SCLC cells.

It is possible that communication occurs between the ER and mitochondria, and this communication may involve Ca^2+^, which plays an important role in conferring cell sensitivity to apoptosis. The rapid cytosolic release of Ca^2+^ from the ER under stress disturbed the morphology and function of the mitochondria, resulting in the initiation of an intrinsic apoptotic pathway. A number of drugs have been reported to induce apoptosis in various NSCLC cells through both mitochondrial and ER-associated pathways, including iridium (III) complex in A549 cells [39], catechin-7-O-xyloside in H1299 cells [40], curcumin in H460 cells [41], and furanodiene in 95-D cells [42].

1. Apoptosis did not occur through the death receptor (DR)-induced caspase activation pathway (extrinsic caspase-dependent pathway). It was reported that the cross-linking of DR with its natural ligand (FasL or Trail) induced the activation of caspase-8 and then caspase-3, followed by cleavage of target proteins, leading to apoptosis [43]. It was reported that chalcone 2′-hydroxy-4′,5′-dimethoxychalcone activated the DR pathway and led to apoptosis in NSCLC H157, H460, H1792, H358 and H322 M cells [44]. In this study, the H446 cells treated with EVO showed no change in the protein expression of Fas, Trail or caspase-8; in the case of H1688 cells treated with EVO, the level of caspase-8 protein expression was not changed. Therefore, we concluded that EVO did not trigger apoptosis through the DR-induced pathway. On the other hand, although the protein expression of caspase-8 in EVO-treated cells was unchanged compared to controls, the activity of caspase-8 increased by ∼120% (EVO treatment for 24 h), ∼215% (48 h), and ∼200% (72 h) compared to controls. The reason that the caspase-8 activity increased after treatment with EVO for certain periods is still not clear, and further study is needed to address this question.

It has been previously reported that some drugs exert anti-cancer effects by inducing not only cell cycle arrest but also apoptosis via intrinsic caspase-dependent pathways in different NSCLC cells. For example, ent-11α-Hydroxy-15-oxo-kaur-16-en-19-oic-acid induced G2-phase arrest and apoptosis via the mitochondria-mediated pathway in A549 [23]; capilliposide induced S-phase arrest and apoptosis via the mitochondria-mediated pathway in A549, H1299 and H460 cells [45]; dioscin induced S-phase arrest and apoptosis via the mitochondria-mediated pathway in A549, H460 and H446 cells [46]; a podophyllotoxin derivative induced M-phase arrest and apoptosis via the ER stress pathway in A549 cells [47]; alkylphenols induced G1 arrest and apoptosis via the ER stress pathway in A549 and H1299 cells [48]; and OSU03013 (a derivative of celecoxib) induced G1 arrest and apoptosis via the ER stress pathway in CL1-1 and H1435 cells [49]. To date, there has been no report of a drug that simultaneously induces cell cycle arrest and apoptosis in SCLC cells via the mitochondria-mediated and ER stress pathways. For the first time, we report that EVO induced G2/M arrest and apoptosis via both the mitochondria-mediated and ER stress pathway in H446 SCLC cells.

In conclusion, EVO exerted significant suppressive effects on the growth of human H446 and H1688 SCLC cells by inducing cell cycle arrest at G2/M phase and subsequent mitochondria-mediated and ER stress-induced caspase-dependent apoptosis. Our findings suggested that EVO is a promising, novel and potent antitumor drug candidate for small-cell lung cancer. The cell cycle, mitochondria and ER stress are rational targets for the future development of an EVO (therapeutic drug) delivery system.
